# Utility of qSOFA score in identifying patients at risk for poor outcome in *Staphylococcus aureus* bacteremia

**DOI:** 10.1186/s12879-019-3770-4

**Published:** 2019-02-13

**Authors:** Emi Minejima, Vanessa Delayo, Mimi Lou, Pamela Ny, Paul Nieberg, Rosemary C. She, Annie Wong-Beringer

**Affiliations:** 1 Department of Clinical Pharmacy, University of Southern California School of Pharmacy, 1985 Zonal Ave, Los Angeles, CA 90089 USA; 2 Department of Pharmacy, Huntington Hospital, 100 W. California Blvd, Pasadena, 91105 USA; 3 Department of Medicine – Infectious Diseases, Huntington Hospital, 100 W. California Blvd, Pasadena, 91105 USA; 4 Department of Pathology, Keck School of Medicine, Los Angeles, 90089 USA

**Keywords:** *S. aureus*, Bacteremia, Mortality, Sepsis-3, qSOFA

## Abstract

**Background:**

The prognostic capability of the quick Sequential Organ Failure Assessment (qSOFA) bedside scoring tool is uncertain in non-ICU patients with sepsis due to bacteremia given the low number of patients previously evaluated.

**Methods:**

We performed a retrospective cohort study of adult hospitalized patients with *Staphylococcus aureus* bacteremia (SAB). Medical charts were reviewed to determine qSOFA score, systemic inflammatory response syndrome (SIRS) criteria, and Pitt bacteremia score (PBS) at initial presentation; their predictive values were compared for ICU admission within 48 h, ICU stay duration > 72 h, and 30-day mortality.

**Results:**

Four hundred twenty-two patients were included; 22% had qSOFA score ≥2. Overall, mean age was 56y and 75% were male. More patients with qSOFA ≥2 had altered mentation (23% vs 5%, *p* < 0.0001), were infected with MRSA (42% vs 30%, *p* = 0.03), had endocarditis or pneumonia (29% vs 15%, *p* = 0.0028), and bacterial persistence ≥4d (34% vs 20%, *p* = 0.0039) compared to qSOFA <2 patients. Predictive performance based on AUROC was better (*p* < 0.0001) with qSOFA than SIRS criteria for all three outcomes, but similar to PBS ≥2. qSOFA≥2 was the strongest predictor for poor outcome by multivariable analysis and showed improved specificity but lower sensitivity than SIRS ≥2.

**Conclusions:**

qSOFA is a simple 3-variable bedside tool for use at the time of sepsis presentation that is more specific than SIRS and simpler to calculate than PBS in identifying septic patients at high risk for poor outcomes later confirmed to have *S. aureus* bacteremia.

## Background

Sepsis is a significant cause of critical illness worldwide with an increasing incidence afflicting over 500,000 cases/year [1] and mortality rates up to 80% [2, 3]. Improved understanding of the pathobiology of sepsis has highlighted the heterogeneity in the host immune response in sepsis, prompting a need to redefine and update the clinical criteria to characterize the syndrome [4–7].

Sepsis is currently defined as a life-threatening acute organ dysfunction secondary to a dysregulated host response to infection (Sepsis-3) [6]. Previously, sepsis was defined as meeting ≥2 systemic inflammatory response syndrome (SIRS) criteria plus suspected infection. However, the SIRS definition was criticized for its lack of sensitivity; a recent study showed that 1 in 8 patients admitted to the intensive care unit (ICU) with severe sepsis did not meet the requisite minimum of 2 SIRS criteria to fulfill the sepsis definition [8]. Thus, to improve the identification of patients at risk for clinical deterioration from infection, Sepsis-3 recommends a quick scoring system, quick sequential organ failure assessment score (qSOFA), comprised of 3 elements assessed at the bedside (altered mental status, respiratory rate, and systolic blood pressure) and without the need for laboratory tests. Patients who show evidence of 2 out of 3 elements are considered positive for risk of clinical deterioration and therefore should prompt clinicians for investigation of organ dysfunction, initiation or escalation of therapy, and consideration of transfer of care to the ICU.

*Staphylococcus aureus* is the most common bacterial cause of sepsis in ICU patients [9]. *S. aureus* bacteremia (SAB) affects an estimated 50/100,000 population, with an overall mortality rate of 19–57% in adults [10]. Despite receipt of the standard treatment, persistent bacteremia occurs in one-third of patients beyond 7 days [11, 12], which can lead to metastatic complications, relapse, prolonged hospitalization, and increased mortality [11, 13–16].

Since Sepsis-3, the prognostic utility of qSOFA for in-hospital mortality has been prospectively validated in patients presenting to the emergency department with suspected infection [17], as well as retrospectively evaluated [18–26] but only a small number of evaluated patients had documented bacteremia. Thus, our study aims to evaluate non-ICU patients presenting with sepsis who later were confirmed to have *S. aureus* bacteremia for 1) differentiating clinical characteristics based on qSOFA score and 2) comparison of the predictive performance of qSOFA to SIRS criteria and Pitt bacteremia score (PBS) as a prognostic tool to identify those at high risk for poor outcome.

## Methods

This was a 4-year retrospective cohort study conducted at three university-affiliated medical centers (600-bed community teaching, 600-bed county teaching, and 400-bed academic) in Los Angeles County, California. The study protocol was approved by the institutional review boards at each study site (University of Southern California and Quorum Review) and as the study was retrospective, informed consent was waived. Microbiology reports were screened for all patients with at least one positive blood culture for *S. aureus* between 2012 and 2016. Inclusion criteria were: 1) age ≥ 18 years, 2) receipt of ≥48 h of effective anti-staphylococcal therapy, 3) initiation of effective anti-staphylococcal therapy within 48 h from the time the first positive blood culture was drawn, 4) monomicrobial growth in the blood culture, and 5) not admitted to the ICU prior to or at onset of bacteremia. Patients with receipt of ≤48 h of effective therapy were excluded as assessment of therapy on mortality outcome was unlikely to be contributory to the antibiotic therapy given.

Medical charts were reviewed for relevant demographics including comorbidities, laboratory, radiographic data, surgical and antimicrobial management, and clinical data. Extracted data were entered into a secure database, the Research Electronic Data Capture software hosted by University of Southern California.

### Study definitions

qSOFA score was calculated for each patient using the worst value documented in the chart within the 24 h period of when the first positive blood culture for *S. aureus* was drawn for three clinical variables worth 1 point each: systolic blood pressure ≤ 100 mmHg, respiratory rate ≥ 22 breaths/min, and Glasgow coma score < 15 or altered mental status noted by the treating physician [6]. qSOFA score of ≥2 points was used as the prognostic cutoff value in predicting clinical deterioration (ICU admission within 48 h and ICU stay > 72 h) and death within 30 days. PBS was calculated for each patient at the onset of bacteremia to characterize the severity of illness based on 5 variables which included temperature, blood pressure, need for mechanical ventilation, evidence of cardiac arrest, and mental status [14]. The SIRS criteria and severity of sepsis were also evaluated according to the 2012 surviving sepsis campaign definitions [27].

The source of bacteremia was divided relative to risk of mortality: low (< 10%), intermediate (10–20%), and high (> 20%) as previously defined [28]. Antibiotic therapy was considered effective if antimicrobial sensitivity was documented. Early clinical response was evaluated on day 4 (72–96 h after initiation of effective antibiotic therapy) and determined as success or failure. Success was complete or partial resolution (objective signs of improvement without complete resolution) of fever, leukocytosis, local signs of infection, and clearance of blood cultures. Failure was defined as persistent growth of blood cultures and/or worsening in objective signs and symptoms of infection, including lack of resolution of fever, worsening or no improvement of leukocytosis, and/or lack of improvement of local signs of infection as documented by the treating physician. Mortality was defined as death occurring within 30 days from date of when the first positive blood culture was drawn.

### Data analysis

Patients were grouped by high (≥ 2 points) or low (< 2 points) qSOFA scores as defined by Sepsis-3 criteria [6] and compared for demographics, clinical presentation and management, and outcomes. Endpoints for evaluation of prognostic capability were ICU admission within 48 h of SAB presentation, length of ICU stay > 72 h during the course of SAB, and 30-day mortality. The prognostic value of meeting at least two SIRS criteria [27] was compared to qSOFA score of ≥2. The positive predictive value (PPV), negative predictive value (NPV), sensitivity, and specificity of qSOFA score of ≥2, SIRS score ≥2, and PBS score ≥2 to predict each study endpoint were calculated. PBS score ≥ 2 was chosen based on the study by Hill et al. which showed a doubling of relative risk for mortality when PBS scores were 2 compared to patients with scores of < 2 in SAB [14]. A receiver operating characteristic (ROC) curve was created to calculate the corresponding area under the ROC (AUROC). Secondary endpoints included SAB outcomes of day 4 clinical failure and persistence of bacteremia past 4 days.

Descriptive analyses were conducted using Wilcoxon rank sum tests or student *t* test for continuous data and chi-square or Fisher’s exact test for categorical data. Univariate followed by multivariable logistic regression analysis were performed to determine the predictors of each outcome after controlling for age, gender, and source risk category. Only variables found to be significantly different between groups by univariate analysis were included in the multivariable logistic regression analysis for each primary endpoint. A *p* value < 0.05 was considered significant. Statistical analyses were performed using GraphPad Prism v4.0 (San Diego, CA, USA) or SAS version 9.4 (SAS Institute, Cary, NC).

## Results

### Study population

A total of 623 hospitalized patients with growth of *S. aureus* in a blood culture were screened; 402 patients met inclusion criteria. Two hundred and twenty-one patients were excluded for the following reasons: 3 patients were < 18 years old, 60 patients received < 48 h of effective antimicrobial therapy, 9 patients were initiated on antibiotics > 48 h from the first positive blood culture, 70 patients had polymicrobial blood cultures, 17 patients had incomplete medical charts, and 62 patients were admitted in the ICU > 24 h prior to the onset of bacteremia. Overall, demographics showed the mean age of 56 years, 75% were male, and 87% had community-onset bacteremia (Table 1). Nearly one quarter (22%, 90/402) of included patients had qSOFA scores ≥2 and were considered the high qSOFA group. Among those who acquired nosocomial SAB (*n* = 53), high qSOFA patients had longer duration of hospitalization prior to onset of bacteremia compared to the low qSOFA group (median 20 vs 9 days, *p* = 0.018). Regardless of qSOFA score, about half the patients (high 50% vs low 53%, *p* = 0.63) had history of three or more comorbidities as documented by the treating physician in the medical chart. Notably, a higher proportion of low qSOFA patients (10% vs 2%, *p* = 0.023) had no pre-existing comorbid condition per physician documentation in the medical chart. One third of patients had presence of hardware at the onset of SAB (high 33% vs low 30%, *p* = 0.53). Overall, MSSA was the predominant (67%) infecting pathogen. The high qSOFA group were more likely to be infected with MRSA (42% vs 30%, OR 1.72, 95% CI 1.06–2.79, *p* = 0.03) and twice as likely to have a high-risk source of bacteremia (29% vs 15%, OR 2.39, 95% CI 1.37–4.15, *p* = 0.0028). The high-risk sources were primarily endocarditis/endovascular source (16%) or pneumonia (11%) in the high qSOFA group. Skin and soft tissue (20%) and osteoarticular (20%) were the most common sources in the low qSOFA group.

**Table 1:** Baseline Demographics comparing patients with high qSOFA score vs low qSOFA scores

Characteristics	High qSOFA *n* = 90 (%)	Low qSOFA *n* = 312 (%)	*p* value
Age, yr. ^a^	56.5 ± 13.99	55.2 ± 15.1	0.66
Male	60 (67)	241 (77)	0.053
Residence Prior to Admission			
Home	66 (73)	240 (77)	0.81
Skilled Nursing Facility	5 (6)	11 (4)	
Other hospital/rehab center	9 (10)	30 (10)	
Homeless	10 (11)	31 (10)	
Comorbid conditions			
None	2 (2)	30 (10)	0.02
Diabetes Mellitus	35 (39)	142 (46)	0.28
End stage renal disease on dialysis	16 (18)	51 (16)	0.75
Cirrhosis	11 (13)	32 (11)	0.52
Cardiovascular disease ^b^	40 (44)	170 (54)	0.096
Immunosuppressed ^c^	15 (17)	37 (12)	0.28
≥ 3 comorbid conditions	45 (50)	165 (53)	0.63
Race/Ethnicity			0.18
Caucasian	28 (33)	92 (30)	
Asian	14 (16)	26 (8)	
African American	10 (12)	34 (11)	
Hispanic	32 (37)	137 (44)	
Other	2 (2)	21 (7)	
History of Intravenous Drug Use	13 (15)	32 (10)	0.26
History of *S. aureus* infection	14 (16)	57 (18)	0.08
History of IV vancomycin therapy	12 (13%)	45 (15%)	0.79
Community-onset SAB	77 (86)	272 (87)	0.72
Microbial characteristics			0.03
MSSA	52 (58)	219 (70)	
MRSA	38 (42)	93 (30)	
Source Risk Category ^d^			0.0036
Low risk	20 (22)	64 (21)	
Intermediate risk	43 (48)	202 (65)	
High risk	26 (29)	46 (15)	
Study site			0.43
County teaching hospital	64 (71)	205 (66)	
Academic hospital	11 (12)	35 (11)	
Community teaching hospital	15 (17)	72 (23)	

^a^ mean ± standard deviation; ^b^ Cardiovascular disease includes hypertension, dyslipidemia, congestive heart failure, coronary artery disease; ^c^ Immunosuppressed: malignancy, recent chemotherapy, chronic steroid use (prednisone ≥20 mg/day or equivalent); SAB = *S. aureus* bacteremia; ^d^ Sources of infection considered low risk were intravascular (IV) catheters, urinary tract infection, ear-nose-larynx, gynecologic, and several manipulation-related sources; intermediate risk were osteoarticular, soft-tissue, and unknown sources; and high risk were endovascular, lower respiratory tract, intra-abdominal, and central nervous system foci

### Clinical presentation and management

The high qSOFA group had a sicker presentation at onset of bacteremia with higher proportion of patients with PBS ≥2 points (69% vs 12%, *p* < 0.0001). (Table 2) Nearly all (98%) patients in the high qSOFA group met SIRS criteria for sepsis compared to 74% in the low qSOFA group (*p* < 0.0001). In the high qSOFA group, respiratory rate ≥ 22 was the most frequent qSOFA criteria met (85% vs low qSOFA 24%, *p* < 0.0001), followed by systolic blood pressure ≤ 100 mmHg (68% vs low qSOFA 18%, *p* < 0.0001).

**Table 2:** Clinical Presentation at onset of *S. aureus* bacteremia

Clinical Presentation	High qSOFA *n* = 90 (%)	Low qSOFA *n* = 312 (%)	*p* value
Pitt Bacteremia Score ^a^	2 (0, 3)	0 (0, 1)	< 0.0001
Score ≥ 2	57 (69)	37 (12)	< 0.0001
Score ≥ 4	19 (23)	7 (2)	< 0.0001
SIRS criteria			
Sepsis	88 (98)	232 (74)	< 0.0001
Severe sepsis	59 (66)	86 (28)	< 0.0001
Septic shock	25 (28)	3 (1)	< 0.0001
No sepsis	2 (2)	80 (26)	
Presenting symptoms			
Fever	35 (39%)	100 (32%)	0.25
Pain	28 (31%)	155 (50%)	0.0018
Altered mental status	21 (23%)	16 (5%)	< 0.0001
Shortness of breath	10 (11%)	19 (6%)	0.11

^a^ median (IQR), data available for 83 patients in High qSOFA group and 301 patients in the Low qSOFA group

Overall, combination of vancomycin with a beta-lactam antibiotic for empiric therapy was the most common therapy administered (high 63% vs low 53%) though vancomycin monotherapy was more frequent in the low qSOFA group (29%) compared to the high qSOFA group (12%). Of the patients infected with MSSA, 76% (205/271) were treated with an anti-staphylococcal beta-lactam agent for definitive therapy, while MRSA bacteremia patients were treated with vancomycin in 59% (77/131) and daptomycin in 27% (36/131) as definitive therapy. There was a trend towards more patients in the high qSOFA group (78%) receiving effective therapy on or before the first day of positive blood culture (vs low 68%, *p* = 0.088). Duration of effective therapy during hospitalization was significantly longer in the high qSOFA group compared to the low qSOFA group (high 14 days vs low 8.5 days, *p* = 0.0011). Similar rates of patients in both groups received Infectious Disease (ID) consultation (high 52% vs low 58%, *p* = 0.33) with the same median of 2 days for time to receive consultation between the groups. Source control procedure for the SAB management was also similar in rates in both groups (high 40% vs low 49%, *p* = 0.12) with a median of 2 days to perform the procedure (high 2 days (IQR 1, 4.75) vs low 2 days (IQR 1, 4), *p* = 0.41).

### Prognosis and outcome

Patients with high qSOFA score had significantly worse prognosis compared to those in low qSOFA group: ICU admission at any point after the onset of SAB was more frequent (64% vs 21%, *p* < 0.0001), more patients were transferred into the ICU within 48 h of onset of SAB (57% vs 16%, *p* < 0.0001), overall duration of ICU stay was prolonged by 2 days (median, *p* = 0.011), and a significantly higher rate of ICU stays of > 72 h (48% vs 12%, *p* < 0.0001). (Table 3) In addition, the high qSOFA group had higher rate of persistently positive blood cultures for *S. aureus* despite receipt of ≥4 days of effective therapy (34% vs 20%, *p* = 0.0039), higher rate of 30-day mortality (19% vs 3%, *p* < 0.0001), and a longer length of stay after the onset of SAB by 5 days (median) compared to the low qSOFA group (*p* = 0.001). Among patients with initial high qSOFA scores, 49% experienced clinical failure at day 4 and were 11 times more likely to die than those showing early clinical response (34%, 15/44 vs 4%, 2/45, *p* = 0.0004; OR 11.12, 95% CI: 2.36–52.35). On the contrary, the mortality rate was only 8% (7/83) in the low qSOFA group who experienced early failure. Among those who died (*n* = 26), median time to death from initial positive blood culture was 10 days and 18 days for the high and low qSOFA group, respectively (*p* = 0.26). In a sub-analysis of patients in the high qSOFA group, those who died (*n* = 17) vs survived (*n* = 73) were older (mean age: 61.7y ± 3 vs 55.6y ± 1.7, *p* = 0.11), had more comorbid conditions (3 or more: 71% vs 45%, p = 0.1), received ID consultation (65% vs 49%, *p* = 0.29), and infection with MRSA (53% vs 40%, *p* = 0.42) but the differences observed did not reach statistical significance. However, rate of patients with ICU admission (88% vs 45%, *p* = 0.002) and need for vasopressors (88% vs 22%, *p* < 0.0001) were significantly more frequent in those who died vs survived.

**Table 3:** Comparison of Clinical Outcomes of *S. aureus* bacteremia by qSOFA score

Clinical Outcomes	High qSOFA *n* = 90 (%)	Low qSOFA *n* = 312 (%)	*p* value
ICU admission	58 (64)	67 (21)	< 0.0001
ICU admission within 48 h of first positive culture	51 (57)	50 (16)	< 0.0001
Need for vasopressors	31 (35)	17 (6)	< 0.0001
Duration of ICU stay, days^a^	5 (2, 12.3)	3 (1, 7)	0.01
Duration of ICU stay > 72 h	43 (48)	38 (12)	< 0.0001
Day 4 Success	45 (50)	228 (73)	< 0.0001
Died	2 (4)	2 (1)	0.07
Day 4 Failure	44 (49)	83 (27)	< 0.0001
Died	15 (34)	7 (8)	0.0003
Microbial Persistence on day 4	31 (34)	62 (20)	0.0039
Died	10 (32)	3 (5)	0.0007
Initial GCS < 15	54/89 (61)	31/306 (10)	< 0.0001
Died	13 (24)	2 (6)	0.04
Initial SBP ≤ 100 mmHg	57/84 (68)	52/297 (18)	< 0.0001
Died	12 (21)	3 (6%)	0.026
Initial RR ≥ 22 breaths per minute	72/85 (85)	71/298 (24)	< 0.0001
Died	14 (19)	4 (6)	0.021
30-day mortality	17 (19)	9 (3)	< 0.0001
Total Hospital LOS, days ^a^	16 (8, 32)	10 (6, 19)	< 0.0001
LOS after first positive culture, days ^a^	14 (7.8, 23)	9 (6.25, 17)	0.001
Disposition of survivors	*N* = 73	*N* = 303	0.92
Home	48 (66)	198 (65)	
Skilled nursing facility	12 (16)	47 (16)	
Outside Hospital	4 (5)	23 (8)	
Rehab center	7 (10)	23 (8)	
Homeless/jail	2 (3)	12 (4)	

*ICU* intensive care unit, *LOS* length of stay, *GCS* glasgow coma scale score, *SBP* systolic blood pressure, *RR* respiratory rate; ^a^ median (IQR)

### Predictive performance of qSOFA, SIRS, and PBS clinical criteria

**Fig. 1:**
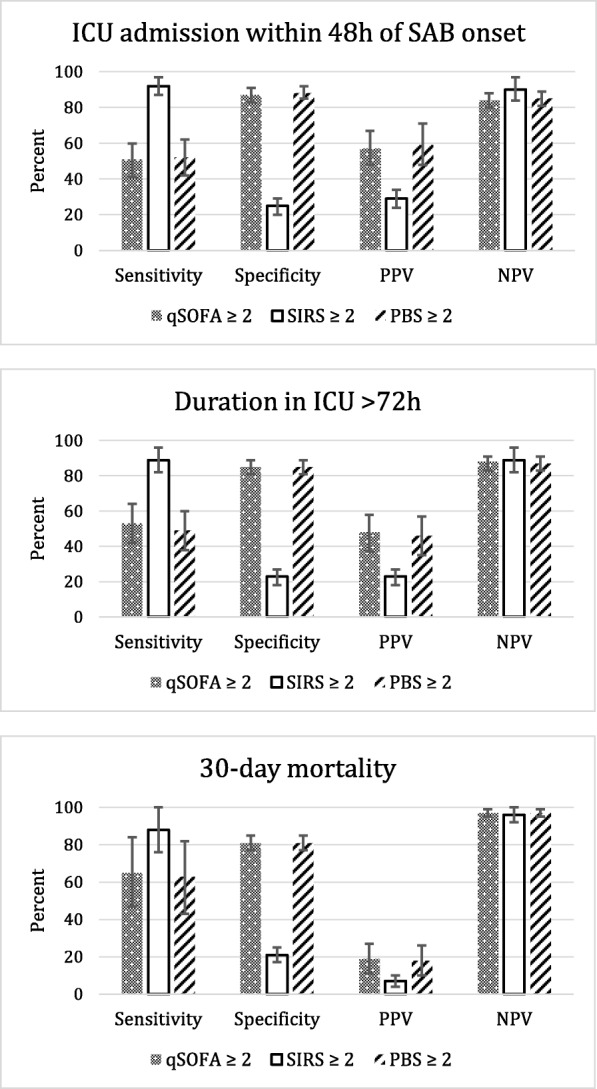
Sensitivity, specificity, positive predictive value, and negative predictive value of scoring systems to predict outcomes. Error bars represent 95% confidence intervals; PBS = Pitt Bacteremia Score

The predictive performance of qSOFA was compared to SIRS and PBS criteria and is shown in Fig. 1. qSOFA had a high specificity and negative predictive value with moderate to poor sensitivity and positive predictive values for all three endpoints, while the reverse was shown for SIRS criteria. The AUROC was significantly higher for qSOFA ≥2 compared to SIRS ≥2 for ICU admission within 48 h of onset of bacteremia [0.7 (95% CI 0.65–0.75) vs 0.58 (95% CI 0.54–0.62)], ICU stay longer than 72 h [0.7 (95% CI 0.64–0.76) vs 0.56 (95% CI 0.52–0.60)], and 30-day mortality [0.76 (95% CI 0.67–0.86) vs 0.54 (95% CI 0.47–0.62) (all comparisons *p* < 0.0001). The AUROC for qSOFA ≥2 was similar to PBS ≥2 for ICU admission within 48 h of onset of bacteremia [0.7 (95% CI 0.65–0.75) vs 0.7 (95% CI 0.65–0.75), *p* = 1], ICU stay > 72 h [0.7 (95% CI 0.64–0.76) vs 0.67 (95% CI 0.67 (0.61–0.73), *p* = 0.34)], and 30-day mortality [0.76 (95% CI 0.67–0.86) vs 0.72 (95% CI 0.62–0.82), *p* = 0.46]. The most significant factors identified from the univariate analysis were evaluated in a multivariable model and controlled for age, gender, and source risk category. qSOFA scores ≥2 was the most significant predictor of each outcome in the model, with more than 4 times greater risk for poor outcome compared to those with qSOFA < 2 (Table 4). SIRS ≥2 was removed from the model as it was not significant. PBS ≥2 was a significant predictor for all three outcomes but the risk associated with qSOFA ≥2 was approximately two-fold greater for the outcome of duration of ICU stay > 72 h and 30-day mortality.

**Table 4:** Predictors of Outcome by Multivariable Logistic Regression

	ICU admission within 48 h of onset of SAB		Duration of ICU stay longer than 72 h		30-day mortality	
Variable	OR (95% CI)	*p*-value	OR (95% CI)	*p*-value	OR (95% CI)	*p*-value
Age	0.99 (0.97–1.00)	0.10	0.99 (0.97–1.00)	0.13	1.03 (1.00–1.06)	0.08
Gender	1.14 (0.62–2.11)	0.68	1.04 (0.55–1.97)	0.90	0.90 (0.34–2.42)	0.84
Source Risk Category: High vs. Intermediate & Low	2.42 (1.27–4.59)	0.007	2.01 (1.05–3.85)	0.04	0.78 (0.26–2.29)	0.65
qSOFA ≥ 2	4.40 (2.40–8.09)	< 0.0001	4.67 (2.51–8.68)	< 0.0001	6.94 (2.49–19.31)	0.0002
PBS ≥ 2	4.69 (2.54–8.67)	< 0.0001	2.89 (1.53–5.48)	0.001	3.38 (1.25–9.16)	0.02

*ICU* intensive care unit, *qSOFA* quick sequential organ failure assessment, *PBS* pitts bacteremia score, *OR* odds ratio

## Discussion

Sepsis-3 introduces qSOFA as a simple bedside tool for screening patients with suspicion of infection who are at increased risk for clinical deterioration [6]. Our study aimed to retrospectively evaluate the predictive performance of qSOFA scoring system in the setting of *S. aureus* bacteremia which has not been adequately studied previously.

Our findings are consistent with those from prior studies involving other infectious syndromes. Freund et al. prospectively evaluated the prognostic accuracy of qSOFA to predict poor outcomes in patients presenting to the emergency department with suspicion of infection. Similar to our study, they reported 24% of their study population had qSOFA scores ≥2 and that qSOFA had improved predictive performance compared to SIRS with AUROC of 0.73 (95% CI 0.68–0.77) for ICU admission, 0.71 (95% CI 0.66–0.76) for duration in ICU > 72 h, and 0.8 (95% CI 0.74–0.85) for in-hospital mortality [17]. In our study, those with initial qSOFA score of ≥2 were 8 times more likely to have prolonged ICU length of stay (> 72 h) and a fatal outcome when compared to patients with a qSOFA score < 2. We used another severity of illness score, PBS previously validated in SAB to predict mortality [14]. PBS scoring system uses 5 variables, 3 of which overlap with qSOFA (hypotension, mental status, and respiratory status) while the other 2 are temperature and evidence of cardiac arrest; the latter requires laboratory testing. Although similar variables are used in the PBS, parameters such as fever and altered mental status are stratified such that more points are assigned as the measured value is increasingly abnormal, whereas with qSOFA the points are assigned with a simple yes or no. The AUROC was comparable between qSOFA and PBS for each of the predefined outcomes. Considering that both PBS ≥2 and qSOFA ≥2 were shown to significantly predict the three clinical outcomes by multivariable logistic regression analysis, qSOFA would be a more practical tool for use at the bedside given its ease of use compared to PBS.

The goal of the qSOFA scoring system is to identify those at increased risk for clinical deterioration [29], and qSOFA score ≥ 2 had good negative predictive value and specificity in predicting prolonged ICU stay and 30-day all-cause mortality in patients with SAB, similar to prior studies [17, 21, 26]. Inversely, SIRS criteria has high sensitivity but was associated with high rate of false positives. Although qSOFA may lack sensitivity compared to SIRS in capturing patients who died, there were only 9 deaths in the low qSOFA group, of which 7 patients had non-SAB related deaths, including withdrawal of care. As SIRS criteria was criticized for lacking specificity which resulted in over-prescribing of antimicrobials, the increased specificity with qSOFA criteria supports its use to be a favored screening system to identify patients most likely to have poor outcome and therefore needing higher level of care.

MSSA was the predominant pathogen overall. Nonetheless, a significantly higher proportion of patients in the high qSOFA group (42%) was infected with MRSA. Notably, among patients with high qSOFA score, clinical outcomes were worse in those infected with MRSA compared to those with MSSA bacteremia: longer length of hospital stay and microbial persistence beyond 4 days of effective therapy. This is consistent with prior literature showing an association of worse outcomes with MRSA compared to MSSA bacteremia [30]. Despite the outcome difference observed between MSSA and MRSA-infected patients in our study, there was no significant difference seen in MRSA infected patients with high qSOFA scores in terms of initial PBS score, the proportions with high risk sources of SAB (MSSA 25% vs MRSA 34%, *p* = 0.48), and time to initiation of effective therapy compared to MSSA infected patients with high qSOFA scores. It is possible that inherent difference in antimicrobial efficacy and/or indirect immunomodulatory effects between agents used to treat MRSA and those (beta-lactams) available to treat MSSA bacteremia contributed to the outcome difference [30]. Taken together, results from our study adds to the existing data [17, 18, 20] in support of the use of qSOFA as a bedside tool in identifying non-ICU patients presenting with sepsis (including those later confirmed to have bacteremia due to *S. aureus*) for early and aggressive management.

Importantly, we found that in patients with persistent bacteremia, a high qSOFA score at initial presentation was associated with a 9 times higher risk of death than those with a low qSOFA. This finding is consistent with our previously published study in that a lack of early response in *S. aureus* bacteremia was the strongest predictor of treatment failure in a multivariable logistic regression model [31]. A trend towards higher risk of death was observed among those who continued on the same treatment despite lack of early response compared to those who switched to alternative therapy (mortality 38% vs 10%, *p* = 0.13). In addition, every additional day of persistently positive blood culture with *S. aureus*, significantly increases the patient’s risk for mortality [32]. Thus, early recognition of high-risk patients using qSOFA could prompt clinicians to make a timely change in management including performing source control procedures earlier in the course to avoid negative outcomes. As the availability of rapid diagnostic technology has significantly shortened time to organism identification for bloodstream infection cases, early recognition of patients at high risk of poor outcome could allow antimicrobial stewardship teams to increase their vigilance of aiding clinicians to obtain early source control, screen for metastatic complications, obtain ID consultation, and initiate optimal antimicrobial therapy as soon as resistance information is known.

Our study has several limitations. As qSOFA scores were not used to prospectively guide therapeutic management in this study, we could not control for all confounding variables that may have affected the clinical outcomes. It is notable that similar proportion of patients between the high and low qSOFA groups received infectious disease consultation and source control procedure. While this study was conducted in the same geographic region, the number of patients included in this study was relatively large and included diverse patient populations such as the elderly and the medically underserved younger populations (age range 19–89 years). Our study population had a higher proportion of male over female patients which is consistent with prior epidemiologic studies, which found SAB to occur more frequently in male patients [33, 34]. We evaluated mortality within 30-days from onset of SAB, which is a common endpoint used in SAB studies while others employed in-hospital mortality as an endpoint in the sepsis literature. Only one patient in our study died past 30 days (died on day 46) after the onset of SAB. Thus, using an alternative definition of mortality did not affect our main analysis. Although at initial presentation of sepsis, the diagnosis of *S. aureus* bacteremia would not have been known, our study demonstrated that a high qSOFA score measured at initial presentation of sepsis predicted poor outcomes in this large cohort of patients who had confirmed *S. aureus* bacteremia later. Thus, if prospectively applied, qSOFA would likely demonstrate prognostic capability in patients with sepsis due to a variety of infection types, including bacteremia.

## Conclusion

qSOFA is a simple 3-variable bedside tool that is more specific than SIRS and simpler to calculate than PBS in identifying septic patients at initial presentation (later confirmed to have *S. aureus* bacteremia) who are at high risk for poor outcomes. Patients identified with high qSOFA score should receive aggressive management for infection including possible transfer to higher-level of care in the ICU. Future studies should include prospective evaluation of the utility of qSOFA scoring system in guiding antibiotic selection (e.g. need for MRSA coverage) particularly when combining with use of rapid diagnostic platform to confirm diagnosis of *S. aureus* bacteremia.

## Abbreviations

AUROC: area under the receiver operating characteristic
ICU: Intensive care unit
MRSA: Methicillin-resistant *S. aureus*
MSSA: Methicillin-sensitive *S. aureus*
NPV: Negative predictive value
PBS: Pitt bacteremia score
PPV: Positive predictive value
qSOFA: Quick sequential organ failure assessment
ROC: Receiver operating characteristic
SAB: *Staphylococcus aureus* bacteremia
SIRS: Systemic inflammatory response syndrome
